# Survival Outcomes in Palliative Sedation Based on Referring Versus On-Call Physician Prescription

**DOI:** 10.3390/jcm12165187

**Published:** 2023-08-09

**Authors:** Cristina Lojo-Cruz, Juan Mora-Delgado, Víctor Rivas Jiménez, Fernando Carmona Espinazo, Juan-Bosco López-Sáez

**Affiliations:** 1 Internal Medicine and Palliative Care Clinical Management Unit, Hospital Universitario de Jerez de la Frontera, Ronda de Circunvalación S/N, 11407 Jerez de la Frontera, Spain; cristilc2@gmail.com (C.L.-C.); victorjjrivas@hotmail.com (V.R.J.); 2 Internal Medicine and Palliative Care Clinical Management Unit, Hospital Universitario de Puerta del Mar, Avenida Ana de Viya 21, 11009 Cádiz, Spain; ffercares@gmail.com; 3 Internal Medicine and Palliative Care Clinical Management Unit, Hospital Universitario de Puerto Real, Calle Romería 7, 11510 Puerto Real, Spain; juanbosco.lopez@gm.uca.es

**Keywords:** palliative care, terminal care, deep sedation, quality of health care, attitude of health personnel, physician’s role

## Abstract

This study sought to determine the survival duration of patients who underwent palliative sedation, comparing those who received prescriptions from referring physicians versus on-call physicians. It included all patients over 18 years old who died in the Palliative Care, Internal Medicine, and Oncology units at the Hospital Universitario of Jerez de la Frontera between 1 January 2019, and 31 December 2019. Various factors were analyzed, including age, gender, oncological or non-oncological disease, type of primary tumor and refractory symptoms. Statistical analysis was employed to compare survival times between patients who received palliative sedation from referring physicians and those prescribed by on-call physicians, while accounting for other potential confounding variables. This study revealed that the median survival time after the initiation of palliative sedation was 25 h, with an interquartile range of 8 to 48 h. Notably, if the sedation was prescribed by referring physicians, the median survival time was 30 h, while it decreased to 17 h when prescribed by on-call physicians (RR 0.357; 95% CI 0.146–0.873; *p* = 0.024). Furthermore, dyspnea as a refractory symptom was associated with a shorter survival time (RR 0.307; 95% CI 0.095–0.985; *p* = 0.047). The findings suggest that the on-call physician often administered palliative sedation to rapidly deteriorating patients, particularly those experiencing dyspnea, which likely contributed to the shorter survival time following sedation initiation. This study underscores the importance of careful patient selection and prompt initiation of palliative sedation to alleviate suffering.

## 1. Introduction

In the final days of life, patients experience numerous symptoms, and there may come a point where these symptoms are not adequately controlled despite receiving optimal palliative care. In these cases, sedation is considered a palliative therapeutic measure in an end-of-life situation [1]. Palliative sedation in the hours before their death has been and continues to be a subject of controversy in clinical, ethical, legal, and religious aspects [2]. Furthermore, those who are not familiar with the indications and technique of sedation or lack experience in palliative medicine may confuse it with a covert form of euthanasia (formerly known as indirect active euthanasia). Despite the clear difference between palliative sedation and euthanasia, there are still individuals, including healthcare professionals, who are unable to distinguish between both options [3]. The proper training of physicians in this practice will ensure that no patient unnecessarily suffers at the end of life [4]. Well-indicated and administered sedation constitutes good medical practice and, therefore, does not admit conscientious objection [5].

The European Association for Palliative Care (EAPC) defines palliative sedation as the deliberate administration of drugs to relieve refractory suffering through the monitored proportional use of medications intended to reduce consciousness in patients with life-limiting diseases [6]. Palliative sedation serves several essential purposes, including managing refractory suffering, addressing emergency situations with imminent death, facilitating end-of-life weaning from life-sustaining support when refractory suffering is foreseeable, and providing temporary respite when conventional treatment fails to offer sufficient relief within an acceptable timeframe. It is crucial to emphasize that the primary objective of palliative sedation is to alleviate refractory suffering rather than to hasten death. When dealing with refractory psychological symptoms and existential distress, the approach differs significantly from other situations due to the dynamic and idiosyncratic nature of the distress, making it challenging to establish refractoriness accurately. Moreover, the use of pharmacological and non-pharmacological approaches in such cases has minimal adverse effects. Additionally, the presence of psychological distress does not necessarily indicate an advanced state of physiological deterioration. These distinctions underscore the importance of tailored and compassionate care in palliative sedation for managing different aspects of suffering effectively [7].

The ultimate goal of sedation is to protect the patient from distress or suffering, which cannot be achieved without reducing their level of consciousness. It is both a patient’s right and a technique for relieving their suffering, as well as a physician’s duty toward the patient’s well-being [8]. The general framework of decision-making should be followed, and thus the patient has the right to receive sedation but not to indicate it [9].

For a proper indication of palliative sedation, the following should be ensured [10]:

- A careful evaluation of the end-of-life diagnosis, ensuring that the patient is truly in the final stage of their life trajectory, which requires a deep understanding of each individual case and a prognostic assessment using valid scales. When we are still at the beginning of the end-of-life phase, therapeutic efforts are more demanding compared to in other situations, for example, in the last days. Therefore, whenever possible, it is recommended that the physician who indicates sedation be the patient’s regular doctor.
- The presence of refractory symptoms and/or physical or psychological suffering. It may happen that we diagnose a refractory symptom for various reasons:
- It could not be adequately controlled with all possible treatments.
- Those treatments may not be appropriate for the patient’s circumstances.
- The waiting time for their effect to take place is not tolerable for the patient.

According to various sources, palliative sedation is used in a significant number of terminally ill patients, ranging from 5% to 52% [11], regardless of whether they have cancer or other diseases [12]. While some studies report its use in 15% to 35% of patients in the last days of life [13,14,15,16], other single-center studies have shown a percentage lower than 15%. The disparity in these figures could be attributed to the lack of a uniform definition, primarily retrospective research practices, and variations in cultural, religious, and ethical values [17].

In fact, the literature clearly shows how the frequency of sedation in terminally ill patients varies widely among different healthcare institutions and countries. For example, in a national study involving 23 Austrian palliative care institutions, the frequency of sedation ranged from 1% to 54%, with hospitalized patients being the most frequent recipients [18]. Similarly, in Italy, a multicenter study described that 15% of patients receiving home care required palliative sedation, increasing to 21% for hospitalized patients [19]. In Korea, a retrospective study of 8309 oncology patients who died in seven tertiary healthcare institutions showed that the proportion of patients receiving palliative sedation varied from 7% to 50%, depending on the institution and responsible Clinical Management Unit [20].

Delirium is estimated to be the most frequent refractory symptom, occurring in approximately 41–83% of cases, followed by pain (25–65%) and dyspnea (16–59%). Other symptoms that led to the initiation of palliative sedation included seizures (5–25%), vomiting (5–22%), malignant obstruction (15%), and massive bleeding (3–5%) [21,22,23].

In some studies, it was described that 10–14% of patients experienced existential distress [24,25]. Existential distress has been defined as “the feeling that one’s own existence is empty or devoid of meaning”. It is a complex concept without a clear definition [26]. It is not only a problem of definitions but also of conceptions. Physicians are knowledgeable about effective medications to alleviate physical pain but often fail to detect or address emotional suffering. Too often, they rely on medications when they should prioritize compassion and psychological therapies. Therefore, a holistic and early approach to patients with serious and incurable illnesses is crucial [27].

Controversy arises when addressing the refractoriness of these types of symptoms, justifying the indication for palliative sedation. In nearly half of the cases, existential suffering has been identified as a type of suffering related to a person’s spirituality or religious experience [28]. Some studies emphasize the importance of proper treatment for spiritual or religious suffering before resorting to palliative sedation [29]. This should include religious services and specialized psychologists as part of the multidisciplinary team alongside healthcare professionals, who may sometimes feel uncomfortable addressing spiritual or religious issues [30,31]. However, some articles question the feasibility of this treatment for refractory existential symptoms, even after exhausting all other options, specifically due to the lack of physical symptoms and the potential for life-shortening effects [32].

Malignant neoplasms most commonly associated with refractory symptoms include lung cancer [33], gastrointestinal tract tumors [21], head and neck cancers [34], and breast cancer [21], although any type of neoplasm can cause these symptoms. Additionally, prevalent non-oncological diseases such as congestive heart failure [35], chronic obstructive pulmonary disease [36], chronic kidney disease [37], and neurological conditions like amyotrophic lateral sclerosis [38] can also present refractory symptoms.

After being sedated, a patient’s lifespan can range from a few hours to several days. It is common for family members to ask the treating physician about the patient’s life expectancy during this time. Unfortunately, there is limited research available to provide clear answers to this issue. According to the available literature, the average length of time from the start of sedation to death can range from 19 h [39] to 3.4 days [40].

A significant amount of time that patients spend in the hospital is under the supervision of on-call doctors. These physicians play a crucial role in shaping the medical course of patients, and one such decision they may need to make is starting palliative sedation. This procedure is used to relieve a patient’s refractory symptoms and must be reviewed and approved by the attending physician.

The purpose of this study was to determine the length of time patients survived after undergoing palliative sedation when it was properly indicated and administered to ease their suffering. The study aimed to investigate if there was a difference in survival time based on whether the palliative sedation was prescribed by the referring physician or the on-call physician.

## 2. Materials and Methods

This study was a retrospective, observational, and analytical cohort study that followed normal clinical practice. The study included all patients who were over 18 years of age and died in the Palliative Care, Internal Medicine, and Oncology units at the Hospital Universitario of Jerez de la Frontera between 1 January 2019 and 31 December 2019.

The study analyzed several variables including age, gender, presence of oncological or non-oncological disease, type of primary tumor (if applicable), need for palliative sedation, and refractory symptoms. Additionally, the study also looked at patient capacity, the main drugs used to control refractory symptoms, whether the sedation was initiated by the referring or on-call physician, and the length of survival after starting palliative sedation (measured in hours), properly indicated and administered to alleviate unbearable suffering.

In this study, the comparison of quantitative variables with a normal or abnormal distribution was carried out using Student’s *t*-test and the Mann–Whitney U test, respectively. The comparison of categorical variables was performed using the chi-square test. After the initial analysis, a binary logistic regression was conducted to compare survival after the start of palliative sedation (less than 24 h or more than 24 h) based on the other variables described.

The results are presented as the mean ± standard deviation, median (interquartile range), or number (percentage) as appropriate. A *p*-value of less than 0.05 was considered statistically significant.

The study was approved by the Cádiz Research Ethics Committee on 4 October 2022 (protocol code TESIS CLC-2022 and registry number 63.22).

## 3. Results

**Figure 1:**
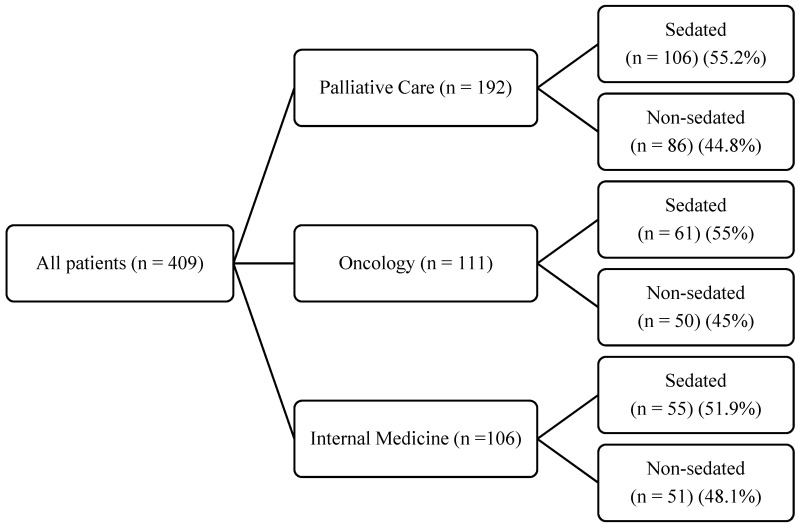
Description of patients who did or did not receive palliative sedation by referring unit.

A total of 409 patients were included in the analysis, of which 222 patients required palliative sedation (54.2%). The patients who required sedation were divided into three categories based on the referring unit: 106 patients (47.7%) were referred from Palliative Care, 61 patients (27.5%) were referred from Oncology, and 55 patients (24.8%) were referred from Internal Medicine (as shown in Figure 1).

The main baseline characteristics of sedated patients are described in Table 1. The patients had a mean age of 69.5 years (median 70 and interquartile range 61–80 years), with 54.5% being men. Regarding their pathology, 24.3% (54 patients) were non-oncological, while 75.7% (168 patients) were oncological. Of the oncological patients, 25.7% (57 patients) had a digestive primary tumor, 23% (51 patients) had a pulmonary primary tumor, 7.2% (16 patients) had a breast primary tumor, 4.5% (10 patients) had head and neck tumors, and 8 patients each had gynecological and urological tumors. Additionally, six patients had central nervous system tumors, four had hematological tumors, four had tumors of unknown origin, three had dermatological tumors, and two had sarcomas.

The main refractory symptoms observed in the study population included dyspnea (45.9%), delirium (14.4%), psycho-existential distress (11.7%), and pain (9.5%) among others. With regard to patient capacity, 19 patients (8.6%) were capable of making decisions while 80 patients (36%) were not, and this information was not recorded in 123 patients (55.4%). For controlling the refractory symptoms, the most commonly used drug was midazolam (33.3%) followed by morphine (8.1%), among others. The specific drugs used were not recorded for 56.8% of patients.

The main refractory symptoms described overall were dyspnea, delirium, and existential suffering, with variations in their distribution among the different units. Dyspnea was the most common symptom among patients receiving palliative sedation under the care of Oncology (50.3%), followed by pain and existential suffering equally (16.5%), delirium (11.6%), and bleeding (3.4%). In the case of Palliative Care, dyspnea was also the most frequent symptom, but to a lesser extent (37.8%), with a higher number of patients experiencing delirium as a refractory symptom (18.9%), ranking existential suffering as the third symptom (15%), and finally pain (8.5%). In relation to Internal Medicine, dyspnea was by far the most common refractory symptom (56.3%), followed by delirium (9.1%) and pain (3.6%). It is worth noting that there were significant differences in the number of patients without a description of the refractory symptom, particularly in the case of Internal Medicine, where this information was not documented in up to 31% of cases (*p* < 0.001).

Regarding the analyzed units (Palliative Care, Internal Medicine, and Oncology), the results showed significant differences among the three groups. Firstly, the median age of patients in the Internal Medicine unit (83 years) was significantly higher than that of Palliative Care (69 years) and Oncology (62 years) (*p* = 0.001). The gender distribution was also different among the groups, with a higher proportion of women in the Internal Medicine unit (60%) compared to Palliative Care (39.6%) and Oncology (42.6%) (*p* = 0.042). The primary pathology causing refractory symptoms was oncological in the majority of patients in the Palliative Care unit (95.3%) and Oncology (100%), while non-oncological diseases were more common in the Internal Medicine unit (10.9%) (*p* < 0.001).

Palliative sedation was indicated by the referring physician in 151 patients (68%), while it was prescribed by the on-call physician in 71 cases (32%). Median survival after the start of palliative sedation was 25 h, with an interquartile range of 8 to 48 h. Specifically, median survival was 30 h (interquartile range of 10 to 50 h) if it was prescribed by the referring physician, while it was reduced to 17 h (interquartile range of 5 to 42 h) in the case of those indicated by the doctor on call.

Significant differences were found among units regarding the prescribing physician of palliative sedation (*p* = 0.037). It was observed that the proportion of patients whose palliative sedation was prescribed by the attending physician was much higher in the Palliative Care unit (82.1%) compared to the Oncology (65.6%) and Internal Medicine units (43.6%). Additionally, significant differences were found in the duration of sedation between the three groups (*p* = 0.012), with a longer duration in patients receiving palliative sedation under the care of Palliative Care (median of 36 h and interquartile range of 14 to 60 h), compared to Internal Medicine (median of 32 h with interquartile range of 8 to 48 h), and to a greater extent, Medical Oncology (median of 8 h with interquartile range of 3 to 24 h).

It was observed, through multivariate analysis, that the prescription of palliative sedation by the doctor on call was significantly associated with a shorter survival time (RR 0.357; 95% CI, 0.146–0.873; *p* = 0.024). Regarding the indication, dyspnea as a refractory symptom was related to a shorter survival time (RR 0.307; 95% CI, 0.095–0.985; *p* = 0.047). Age, gender, type of disease, primary tumor, capacity, or medication were not significantly related to survival time (Table 2).

## 4. Discussion

Our study is noteworthy because it examines the use of palliative sedation across various units and distinguishes it based on the prescribing doctor.

The prevalence of palliative sedation in our cohort was slightly higher than 50%, falling within the upper range (10% to 50%) described in the literature, with an average of 20% to 30% [12,14,41,42]. The higher prevalence in our study may be attributed to several factors. Firstly, there may be differences in clinical practice, with a higher inclination in our healthcare area to use palliative sedation for controlling refractory symptoms. Secondly, our study included a higher proportion of patients with advanced diseases and refractory symptoms. Cultural and educational factors might also contribute, as physicians in our study may hold distinct beliefs and values regarding end-of-life care and palliative sedation. Lastly, the criteria for indicating palliative sedation could vary between studies, and our study might have included more cases where sedation was used for symptom control rather than solely for end-of-life care [43].

The mean age of patients in our study is slightly higher than in other published works, likely because non-cancer patients were not included in those studies. When examining large studies with both cancer and non-cancer patients, the average age aligns with our patients [18]. The proportion of sedated men is higher in our study, consistent with some cohort studies and reviews [13,14], but differs from others where women represent a higher percentage of cases. However, the subtle gender differences in palliative sedation requirements make it challenging to determine whether men or women need it more frequently [15,18]. The observed differences in male patients may be attributed to gender-based variations in symptom severity, disease complications, and attitudes towards palliative sedation [44,45,46].

Regarding the types of primary tumors in cancer patients who received sedation, our findings align with other studies that show that digestive and lung tumors are the most common [39,47]. These high-incidence and lethal tumors often have symptoms that are challenging to manage, which we believe is the main reason for their prevalence in the studies reviewed.

Concerning symptoms, our findings are in line with much of the literature that states dyspnea and delirium are among the most common reasons for palliative sedation [48,49,50]. Among the refractory symptoms described, it is worth noting the complexity in the interpretation of psycho-existential distress as a reason for palliative sedation in some patients, according to the doctor who indicated the sedation. This is present in 36% of the sedations carried out [51]. We report that these cases were only considered after repeated multidisciplinary evaluations, including psychological and/or psychiatric mental health assessments [52]. Existential distress or symptoms are classified as refractory when there are no available methods that are likely to offer suitable relief within an acceptable timeframe and without causing unacceptable adverse effects. This notion of refractoriness can be applied to either a single symptom or state, or to a combination of symptoms or states that collectively create a condition perceived as intolerable by the patient. The determination of refractoriness is a collaborative decision involving the physician (and/or the multi-professional team) and the patient or their legal representative/significant others [6]. Differences in symptom distribution between oncology and non-oncology patients are crucial for understanding their unique needs and challenges. Dyspnea is more common in non-oncology patients, associated with non-malignant conditions [53,54]. Conversely, oncology patients experience higher levels of pain due to the disease and its treatment [25]. Delirium is also more prevalent in oncology patients, possibly linked to neoplasms and treatments [14]. These findings highlight the importance of tailored care approaches for patients with distinct symptom burdens.

Midazolam was the most commonly used medication. The EAPC cites a well-controllable benzodiazepine such as midazolam should be used as a first-line approach [6]. It is the preferred sedative in palliative care and is often combined with other drugs depending on the refractory symptoms [42]. Morphine was the most used drug in conjunction with midazolam, in similar proportions to other studies [14]. It is also an expected result since dyspnea was the most common symptom in our study [55].

The results of the present study indicate significant differences among units regarding the prescriber of palliative sedation. Specifically, the proportion of patients whose palliative sedation was prescribed by the referring physician was considerably higher in the Palliative Care unit compared to the Medical Oncology and Internal Medicine units. One possible explanation is that the Palliative Care unit is more specialized in end-of-life care and has more experience in managing refractory symptoms. As such, the referring physician in the Palliative Care unit may have a better understanding of the patient’s condition and symptom burden, enabling them to make more informed decisions about the use of palliative sedation. Another possible explanation is that the Palliative Care unit emphasizes a team-based approach and greater involvement of referring physicians in decision-making [56]. This approach can foster a greater trust and collaboration between physicians and patients, facilitating better planning of their care according to the course of the disease, which could lead to greater expertise on the part of referring physicians in prescribing palliative sedation [57].

Involving referring physicians in the prescription of palliative sedation offers several important benefits [58]. Firstly, it ensures patients receive consistent and coordinated care throughout their illness, which is particularly crucial for those nearing the end of life as they often require complex and multidisciplinary care [59]. Secondly, it fosters better communication and collaboration among healthcare professionals, allowing the entire team to work together towards a common goal, providing the highest quality of care in the final days [60]. Lastly, involving referring physicians contributes to increased patient and family satisfaction, as it maintains the continuity of care and strengthens the patient-physician relationship [61].

The study offers valuable insights into palliative sedation prescription across different units, revealing a higher involvement of referring physicians in the Palliative Care unit compared to Internal Medicine and Medical Oncology units. There is a significant difference in the prescribing physician of palliative sedation between oncology and non-oncology patients. Referring physicians made the decision to administer palliative sedation in nearly three-quarters of oncology patients (even more so in Palliative Care) compared to less than half of non-oncology patients. Individualized, patient-centered decision-making is crucial, especially for non-oncology patients, considering their overall clinical status, preferences, and care goals [62].

The interval between the initiation and the completion of sedation is similar to other studies [16], while others have observed a longer survival time after its initiation [12,63]. The survival time after palliative sedation can vary due to multiple factors, such as patient-selection criteria, timing of palliative sedation initiation, quality of care provided, level of advance care planning, and the underlying health status of the patient. Our study found that the on-call physician initiated palliative sedation in a smaller proportion of cases compared to other studies, where it represents more than half of the cases [64]. In our study, it only accounted for approximately one-third of the cases. No previous studies have examined survival based on the physician who initiated palliative sedation. Although patients who received palliative sedation initiated by the on-call physician had a shorter survival time compared to those initiated by the referring physician, this should not be interpreted as a result of inadequate treatment. All cases in our study were appropriately indicated and carried out correctly for the sole purpose of symptom relief.

Differences in the duration of palliative sedation initiated by different units were also examined. Patients treated by the Palliative Care unit had the longest duration, while Oncology had the shortest duration. Factors such as diverse approaches to palliative sedation, patient selection, disease trajectories, and symptom profiles could contribute to these differences [65]. Patients with severe metabolic disorders might experience varying sedation durations due to frequent medication adjustments [66]. Understanding patient needs and preferences is crucial for tailoring the sedation approach accordingly. Further research is needed to explore the impact of patient selection on the duration of palliative sedation and develop effective guidelines. The principle of double effect in end-of-life care involves using medications with both intended and unintended effects during palliative sedation [67]. While generally considered ethically permissible, it highlights the importance of carefully considering both the intended and unintended consequences of treatment [54,68]. The principle of double effect in palliative sedation highlights important ethical considerations in balancing desired symptom relief with potential unintended effects, particularly when higher medication doses are needed [69,70]. Patients receiving palliative sedation for dyspnea may have a shorter survival time due to advanced disease progression and the challenging nature of managing dyspnea [71] compared to other symptoms [72,73]. The association between dyspnea as a refractory symptom and a shorter survival time in palliative sedation underscores the importance of considering both the intended and unintended effects of treatment, including ethical principles.

The age differences between oncology and non-oncology patients regarding palliative sedation may be attributed to oncology patients experiencing symptoms and disease progression at a younger age, leading to a higher need for sedation at a younger age. On the other hand, non-oncology patients may have slower-progressing chronic conditions, resulting in a higher average age at sedation [18]. These findings have implications for palliative care management in both patient groups, and further research is needed to explore the reasons behind these differences and optimize the use of palliative sedation for both oncology and non-oncology patients.

The study revealed significant differences in the types of symptoms that led to the initiation of palliative sedation in oncology and non-oncology patients. Non-oncology patients more commonly experienced dyspnea, while pain was more frequent among oncology patients. Additionally, delirium was more prominent in oncology patients compared to non-oncology patients. These findings indicate that the underlying disease and its associated symptoms influence the necessity for palliative sedation [22]. It emphasizes the importance of customizing palliative care interventions to address the specific needs of each patient, considering their distinct clinical presentations and symptom burdens [21].

The main weaknesses of this study are its retrospective nature, which could introduce information and classification biases, although the same criteria were used to collect all variables. Additionally, being a single-center study could be considered a limitation as it did not involve a comparison between different units in multiple hospitals in the area, which would have provided more information and reduced bias induced by the nature of the single-center study. However, this same point could also be considered a strength, as the working methodology of a single hospital, even among different units, allows for precise contrast that eliminates possible biases regarding procedural differences, available resources, or even cultural factors.

## 5. Conclusions

Our study showed a higher frequency of palliative sedation compared to other series, which may be due to the type of patients in our patient population. The distribution of medications used for palliative sedation were similar to those used in other studies. The duration of palliative sedation was shorter or similar to other studies due to the advanced state of the patients.

In many cases, the on-call physician had to intervene with palliative sedation in patients who were in a rapidly deteriorating state, mostly in those experiencing dyspnea. This sudden clinical instability was likely a factor in the shorter survival time after the initiation of palliative sedation.

Overall, the higher frequency and shorter duration of palliative sedation in our study may reflect the advanced state of the patients, particularly those with dyspnea, and the need for a prompt intervention to relieve suffering from the on-call physician. It highlights the importance of careful patient selection and timely initiation of palliative sedation.

Potential future research directions could be around proper training of healthcare professionals, since it is crucial to ensure optimal and quality care at the end of life for patients. In the specific case of palliative sedation, it is important for professionals to be trained to identify and appropriately manage refractory symptoms, respect underlying ethical and legal principles, and accurately document relevant information in the patient’s medical record. Conducting a multicenter study and expanding its scope to encompass other medical specialties that have a significant number of patients who may require palliative care, such as Gastroenterology, Neurology, and Pulmonology, among others, could yield comprehensive insights into the specific circumstances surrounding palliative sedation in these patient populations. This comprehensive approach would facilitate a reassessment of the variables analyzed in the present study and enhance the comprehension of palliative sedation in diverse patient cohorts, each with its own distinct medical contexts.

## Figures and Tables

**Table 1:** Baseline characteristics of patients included in the study.

*n* = 222	Palliative Care (*n* = 106)	Oncology (*n* = 61)	Internal Medicine (*n* = 55)	*p* Value
**Age**, median (Q1–Q3) (years)	69 (61–78)	62 (52–67)	83 (77–89)	**0.001**
**Gender** *n* (%)				**0.042**
Men	64 (60.4%)	35 (57.4%)	22 (40%)	
Women	42 (39.6%)	26 (42.6%)	33 (60%)	
**Disease**, *n* (%)				**<0.001**
Oncologic	101 (95.3%)	61 (100%)	6 (10.9%)	
Non-oncologic	5 (4.7%)	-	49 (89.1%)	
**Main refractory symptom**, *n* (%)				**<0.001**
Not described	21 (19.8%)	1 (1.7%)	17 (31%)	
Dyspnea	40 (37.8%)	31 (50.3%)	31 (56.3%)	
Pain	9 (8.5%)	10 (16.5%)	2 (3.6%)	
Bleeding	-	2 (3.4%)	-	
Delirium	20 (18.9%)	7 (11.6%)	5 (9.1%)	
Psycho-existential distress	16 (15%)	10 (16.5%)	-	
**Capacity**, *n* (%)				**<0.001**
Not described	48 (45.3%)	53 (86.9%)	22 (40%)	
Capable	14 (13.2%)	3 (4.9%)	2 (3.6%)	
Not capable	44 (41.5%)	5 (8.2%)	31 (56.4%)	
**Primary medication**, *n* (%)				**0.049**
Not described	42 (39.6%)	46 (75.4%)	40 (72.7%)	
Midazolam	63 (59.5%)	10 (16.4%)	1 (1.8%)	
Morphine	-	4 (6.6%)	14 (25.5%)	
Levomepromazine	1 (0.9%)	1 (1.6%)	-	
**Prescribing physician**, *n* (%)				**0.037**
Referring physician	87 (82.1%)	40 (65.6%)	24 (43.6%)	
On-call physician	19 (17.9%)	21 (34.4%)	31 (56.4%)	
**Interval between start and end** **of sedation**, median (Q1–Q3) (hours)	36 (14–60)	8 (3–24)	32 (8–48)	**0.012**

**Table 2:** Results on survival after initiation of sedation (less versus more than 24 h).

	Relative Risk	95% Confidence Interval	*p* Value
**Age**	1.028	0.996–1.061	0.087
**Gender** (male)	1.195	0.481–2.906	0.721
**Disease** (oncologic)			1
**Primary tumor**			0.942
Pulmonary	0.515	0.105–2.522	0.413
Breast	0.835	0.318–2.191	0.713
Digestive	2.407	0.367–15.776	0.36
Gynecologic	1.219	0.199–7.481	0.831
Urologic	1.419	0.248–8.105	0.694
Head and neck	1.532	0.198–11.829	0.683
Central Nervous System			1
Sarcoma	0.414	0.029–5.962	0.517
Dermatologic			1
Hematologic	0.337	0.027–4.171	0.396
**Symptom**			0.347
Dyspnea	0.307	0.095–0.985	**0.047**
Pain	0.566	0.129–2.516	0.454
Bleeding			1
Delirium	0.668	0.169–2.638	0.565
Psycho-existential distress	0.779	0.195–3.271	0.755
**Capacity**			0.230
Capable	3.357	0.758–14.861	0.111
Not capable	1.574	0.651–3.808	0.314
**Primary medication**			0.856
Midazolam	1.616	0.692–3.773	0.267
Morphine	0.953	0.126–7.239	0.963
Levomepromazine			1
**Prescribing physician** (on call)	0.357	0.146–0.873	**0.024**

## Data Availability

Data supporting reported results can be shared under request at juanmorainternista@gmail.com.
